# Improved Electromagnetic Interference Shielding Properties Through the Use of Segregate Carbon Nanotube Networks

**DOI:** 10.3390/ma12091395

**Published:** 2019-04-29

**Authors:** Sung-Hoon Park, Ji-Hwan Ha

**Affiliations:** Department of Mechanical Engineering, Soongsil University, 369 Sangdo-ro, Dongjak-Gu, Seoul 06978, Korea; jhwan618@gmail.com

**Keywords:** carbon nanotube, composite, electromagnetic interference shielding, excluded volume

## Abstract

We report the enhanced electromagnetic interference (EMI) shielding properties of hybrid carbon nanotube (CNT) composites consisting of more than two kinds of fillers through the use of segregate conducting networks. An excluded volume was created by micro-sized silica particles that concentrate the CNT network, resulting in improved electrical conductivity and microwave properties. To achieve the optimal dispersion of CNTs and silica particles, high shear force was applied to the pre-cured composite mixture via three-roll milling. Depending on the micro-silica content ratio, we observed improved electrical conductivity and EMI shielding properties. For a quantitative comparison to observe the excluded-volume effects, a CNT composite without micro-silica was measured in parallel with the other sample.

## 1. Introduction

Carbon-based polymer composites have been researched for use in various applications, including structural reinforcement, heating elements, electronic packaging, electromagnetic interference (EMI) shielding, storage capacitors, and so on [1,2,3,4,5,6,7]. Among them, EMI shielding is one of the applications especially important to the military, vehicle, and electronics sectors. Unwanted electromagnetic waves from electronic devices can be absorbed by the human body and interfere with detailed electronic instruments. For example, mobile phone usage is one of the suspected causes of premature cataracts [8]. Current developments in wireless charging and electric motor vehicles are accelerating to produce an increasing amount of electromagnetic pollution. To solve this problem, a polymer-composite containing carbon fillers such as carbon fiber [9], carbon black [10,11,12], and carbon nanotubes (CNTs) [7,13,14,15] have been investigated owing to their flexibility, light weight, chemical corrosion resistance, and relatively good shielding efficiency (SE). A polymer is used as the matrix material in the composite systems, as it has flexibility and electrically insulating properties. With the incorporation of conducting fillers, the low conductivity of the polymer is dramatically increased as conducting networks form. Traditional micro-size conducting fillers have a limit to the filler loading of the polymer due to embrittlement and degradation of the mechanical properties as the amounts of filler used is increased. In addition, intrinsically conducting polymers still require improvement of their mechanical properties [16]. Therefore, a nanosized, high aspect ratio filler is an ideal material to achieve low electrical percolation along with mechanical reinforcement. CNTs are one of the promising fillers for EMI shielding applications, owing to their unique mechanical strength, high aspect ratio (as high as 10^6^), and large surface area (>1300 m^2^/g) [17]. However, there are still many problems facing the commercialization of CNT composites as EMI shielding materials such as bundling and the aggregation of CNTs due to the strong van der Waals attraction between them, resulting in poor dispersion in the polymer. Previously, we have developed an effective CNT dispersion method using three-roll milling, which is effective, especially in high-loading CNTs. Consequently, a maximized EMI SE was attained for a given CNT volume fraction with a very low electrical percolation threshold [13]. 

Recently, hybrid CNT composites comprising more than two types of fillers have been developed to improve the mechanical properties and electrical conductivity [18,19,20]. When a micro-size second filler is added to a CNT-polymer composite system, it can create an excluded volume, resulting in concentrated CNT networks in the polymer matrix. Consequently, in the case of hybrid CNT composites having segregated CNT networks, increased conductivity is achieved compared with two-component CNT-polymer composites [21,22]. Advantages of the segregated CNT composite are material cost reduction for expensive CNTs and mechanical strengthening by adding silica and BaSO_4_ [23]. 

**Figure 1:**
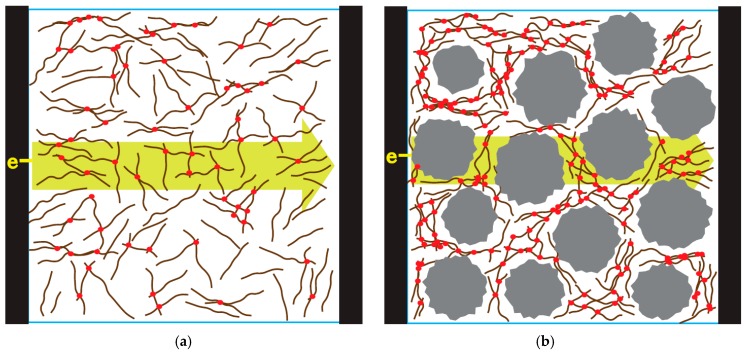
Scheme of (**a**) nanotube composite and (**b**) segregated nanotube composite with secondary micro-silica filler.

In this study, we report enhanced electrical properties and EMI shielding properties through the use of segregate conducting networks. To create an excluded volume and segregated CNT networks, micro-sized silica (SiO_2_) was used. The concept is illustrated in Figure 1. While Figure 1a shows a general, random fiber composite scheme, Figure 1b shows excluded volumes that are created by adding micro-silica particles. Even if the same number of CNTs was used in both cases, the number of contact junctions (red points in Figure 1) between CNTs is different. Consequently, higher electrical conductivity and EMI SE were achieved in the segregated CNT composite. 

## 2. Materials and Methods

For the fabrication of a hybrid CNT composite, multi-walled CNTs approximately 10–15 μm in length and 15 nm in outer diameter (CM250, Hanwa Nanotech Inc., Seoul, Korea) were used for the CNT component, and silica powder of approximately 3–7 μm in particle size (Silbond, Quarzwerke, Bahnhofstraße, Germany) was used as a filler. Polydimethylsiloxane (PDMS Sylgard 184, Dow Corning, Midland, MI, USA) was used as the polymer matrix. To optimize the dispersion conditions of CNTs and silica in a high-viscosity polymer matrix, a paste mixer (Daehwa, PDM-1k, Seoul, Korea) and a three-roll mill (Intech Inc., Gyeonggi-do, Korea) were used. The PDMS part A (elastomer base) and part B (curing agent) were mixed in a 10:1 ratio, following which the CNTs/silica were pre-mixed using the paste mixer. These pre-mixed pastes were then milled by three-roll milling for several minutes. By reducing the gap between the rollers of the mill, a higher shear strain was obtained, resulting in efficient de-bundling of the CNTs. After the mixing process, a heat press (Qmesys Inc., Gyeonggi-do, Korea) was used to cure the composite film at 130 °C (30 min) with constant force (1 ton). The final composite films used for electrical measurements and EMI SE measurements were 2 mm thick. 

To determine the CNT and silica dispersion conditions, scanning electron microscopy (SEM: Phillips XL30, North Billerica, MA, USA) was used. To prepare samples that would allow clear visualization of the bulk morphology, CNT composite films were fractured in liquid nitrogen. The fracture surfaces were coated with ~10 nm Au to wick off electrons to enable clear imaging. For electrical conductivity measurements, CNT–composite films were treated with an oxygen plasma (Femto Science Inc., Gyeonggi-do, Korea) to ensure a low contact resistance, followed by sputtering 50 nm of Au/Ti onto the sample to form the electrodes. 

**Figure 2:**
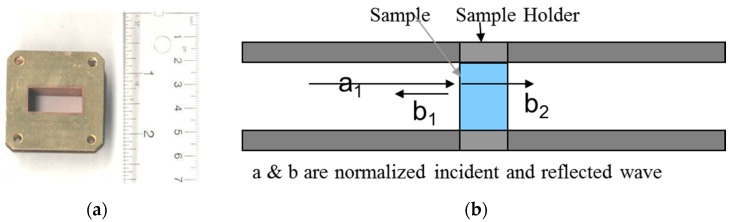
(**a**) Image of the wave-guide holder. (**b**) Scheme of the EMI SE measurement mechanism.

In the case of the resistance, *R* < 1 GΩ, the 4-point resistance method was used to obtain an electrical conductivity (Keithley 2400 source-meter and Keithley 487 picoammeter, Cleveland, OH, USA). For measurement, CNT films were prepared as 10 mm × 2 mm-thick coupons (length: a 50 mm gap for the outer current region, and a 25 mm gap for the inner voltage region). For the EMI SE measurement, uncured CNT paste was inserted into the wave-guide holder as shown in Figure 2a, and then pressed with heat press machine to cure the composite film at 130 °C (30 min) with constant force (1 ton). To avoid air gaps in the sample, press, and release sequences were repeated 3 times at the first. The X-band microwave frequency (8.2–12.4 GHz), employed in military and civil communications, was used for the SE experiments. From a two-port vector network measurement, scattering parameters (*S*_11_ = b_1_/a_1_, *S*_21_ = b_2_/a_1_, where a and b are normalized incident and reflected wave) were obtained as shown in Figure 2b. The EMI shielding effectiveness (SE) was calculated as follows:

Shielding Effectiveness (SE) = −10 log(|−S_21_|^2^) = 10 log (*p_i_*/*p_t_*) (1)

where *p_i_* and *p_t_* are the magnitudes of the incident and transmitted power densities [7]. The EMI SE calibration was conducted using the Thru Reflect Line (TRL) method [24]. In TRL method, thru measurement was conducted by connecting the two waveguide sections (15 cm). For the reflect calibration, an aluminum was placed between two waveguide sections, and line calibration was conducted by insertion of WR-90 waveguide.

## 3. Results and Discussion

**Figure 3:**
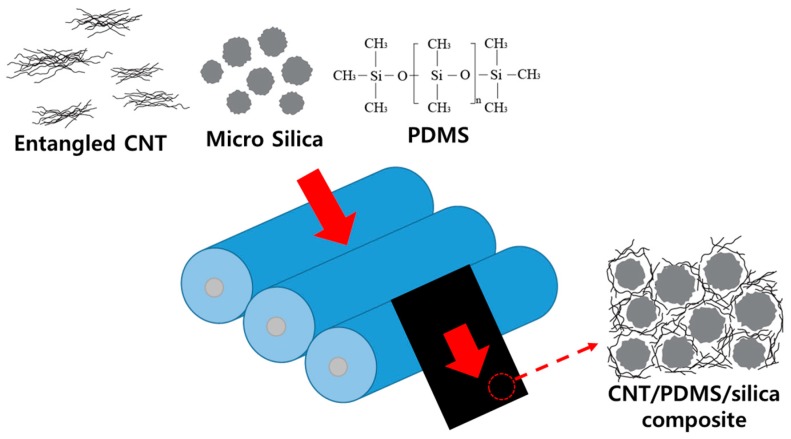
Scheme for the dispersion of highly loaded composites of CNT (carbon nanotube), silica, and PDMS (Polydimethylsiloxane) by three-roll milling. A high shear strain by the three rotating rollers disentangles the CNT and micro-silica.

In general, in our specimens, high aspect ratio CNTs exist in 5–30 μm bundle-type formations due to van der Waals interactions. To maximize the electrical conductivity of the CNT composite at a given CNT loading, each CNT must be de-aggregated and dispersed in the polymer matrix. In addition, a considerable amount of micro-silica should be added to form a segregated CNT network in the polymer matrix. Uniform dispersion is possible through the use of the three-roll milling method, as shown Figure 3, which is optimal for a high-loading mixture-dispersion. High shear forces are created by three rollers moving in opposite directions and with differing speeds, and this can disentangle CNTs. A higher shear force can be obtained by reducing the gap distance between two adjacent rollers. Advantages of this method are uniform shear in the assemblage being mixed in this solvent-free process. In addition, surfactants and chemical functionalization preventing re-aggregation of CNTs are unnecessary due to the high viscosity of the paste. 

**Figure 4:**
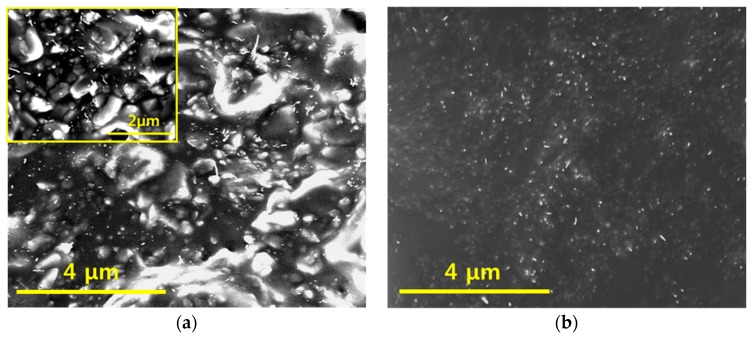
SEM images of (**a**) CNT/micro-silica/PDMS composite and (**b**) CNT/PDMS composite (both 6 wt% CNT). The inset shows a high resolution image of CNT/micro-silica/PDMS composite.

**Figure 5:**
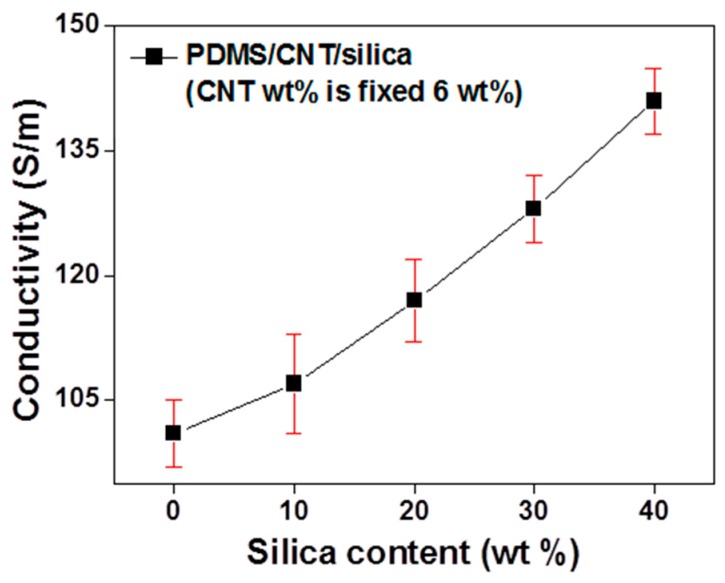
Electrical conductivity of CNT/micro-silica/PDMS composites as a function of micro-silica content. CNT content is fixed at 6 wt%.

For a quantitative comparison, two different types of composites (CNT/micro-silica/PDMS and CNT/PDMS) were fabricated with 5 wt% CNT loading. In both cases, a uniform dispersion of CNT and silica was achieved, as shown Figure 4. Also, to investigate the effect of the secondary silica filler, CNT loading was fixed at 6 wt%. The electrical conductivity was measured with varying micro-silica loading, as shown in Figure 5. Increasing micro-silica content increased the electrical conductivity, which is attributed to the creation of exclude volume in the polymer matrix, similar to that in Figure 1b. Greater micro-sized exclude volumes lead to more concentrated CNT networks, indicating the formation of more contact points between CNTs.

**Figure 6:**
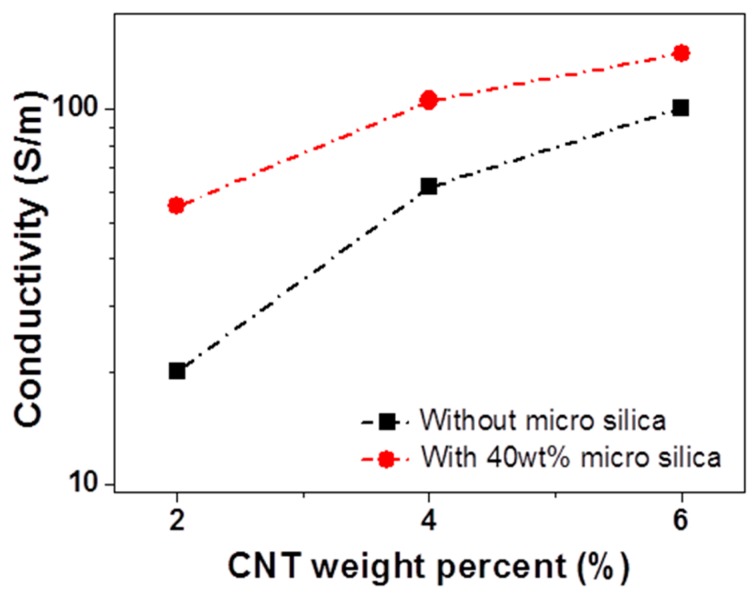
Electrical conductivity of CNT/micro-silica/PDMS and CNT/PDMS composites as a function of CNT content. In the case of the CNT/micro-silica/PDMS composite, silica was fixed at 40 wt%.

To investigate the trend of the conductivity increment, the CNT wt% was increased with a fixed micro-silica content, as shown in Figure 6. One purpose of this study was to make an efficient EMI shielding material. Therefore, all CNT composites were made above the CNT volume-fraction percolation threshold. The secondary filler’s (excluded volume) effect on the electrical conductivity increased when CNT loading was decreased, while its effect at high CNT loading was relatively small. From the power-law trend (σ_DC_ ~ σ_0_(*p* − *p*_c_)^β^) of the fiber-type composite [25], a CNT composite with low CNT loading is more sensitive than a composite with high CNT loading (where σ_DC_ is the conductivity of composite, σ_0_ is a constant parameter, *p* is the volume fraction of filler, *p*_c_ is the percolation threshold, and β is a critical exponent).

Even if high conductivity is not necessary for EMI SE in the high-frequency range, a conductivity related to the conducting network is still the dominant parameter in EMI shielding [26,27,28]. In addition, for effective EMI shielding, conductivity of the composite should be higher than 10 S/m [13]. 

**Figure 7:**
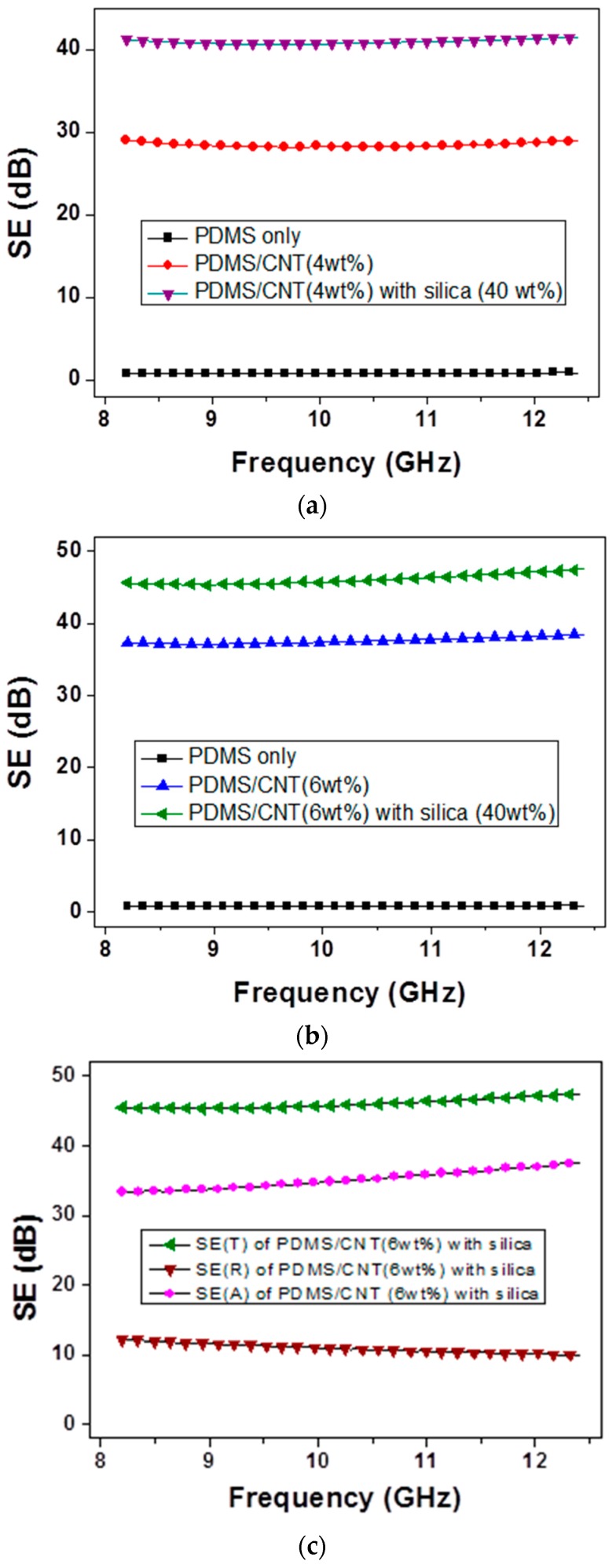
Comparison of EMI SE of CNT/micro-silica/PDMS and CNT/PDMS composites, (**a**) with 4 wt% CNT, and (**b**) with 6 wt% CNT. (**c**) Total SE of CNT/micro-silica/PDMS (6 wt% CNT) can be decomposed into contributions from absorption, SE(A) and reflection, SE(R).

The EMI SE of CNT/silica/PDMS and CNT/PDMS composites in the X-band range were measured, with results shown in Figure 7. The EMI SE of the CNT/PDMS composite was about 30 dB, while a higher SE (40 dB) was attained for the CNT/silica/PDMS composite (CNT content in this case was fixed at 4 wt%). In contrast, in the case of 6 wt% CNT loading, the EMI SE of the CNT/PDMS composite was about 38 dB, while a higher SE (46 dB) was attained for the CNT/silica/PDMS composite, as shown Figure 7b. In both cases, segregated CNT networks in a polymer matrix enhanced the EMI SE, which is a similar trend as that seen with the conductivity behavior. The increment of SE is relatively greater in the “4 wt% CNT” composite (10 dB) than in the “6 wt% CNT” composite (8 dB). Saturation behavior has a similar power law characteristic. For a further understanding of EMI shielding, the measured total SE(*T*) of the composite is divided into the reflection component (*R*) and the absorption component (*A*) as follows [7,24]:

SE(*T*) = SE(*R*) + SE(*A*) (2)

where SE(*R*) = −10 log(1 − *R*) and SE(*A*) = −10 log[*T*/(1 − *R*)]. *R*, *A* and *T* were obtained from the *S* parameter as follows:

*T* = |*S*_21_|^2^, *R* = |*S*_11_|^2^, and *A* = 1 − |*S*_11_|^2^ − |*S*_21_|^2^. (3)

It was seen that absorption-dominated shielding was observed in Figure 7c. In the figure, the SE(A) of CNT/silica/PDMS (6 wt% CNT) composite is higher than SE(R) indicating the microwave absorption is more important parameter in EMI shielding at X-band. This trend regarding the relatively high conductivity composite case is similar to that observed in our previous work [7]. In a further investigation, multi-layer composites having different compositions and contents (by coupling several layers) are planning to enhance the EMI SE and microwave absorption [29,30].

## 4. Conclusions

In summary, we have enhanced the EMI shielding properties of a CNT composite through the use of segregated CNT networks with the assistance of micro-silica particles. Micro-size silica particles create an excluded volume in the polymer matrix, resulting in concentrated CNT network paths. Consequently, the electrical conductivity increased, which is attributed to more CNT–CNT contact points. These advantages can be applied to improve EMI shielding properties. Successful EMI SE values were obtained in this work. Our proposed scheme of using a segregated CNT network with our results could be used as a strong design guideline for EMI shielding materials.
